# Prevalence and risk factors for cancer of the uterine cervix among women living in Kinshasa, the Democratic Republic of the Congo: a cross-sectional study

**DOI:** 10.1186/s13027-015-0015-z

**Published:** 2015-07-15

**Authors:** Catherine Ali-Risasi, Kristien Verdonck, Elizaveta Padalko, Davy Vanden Broeck, Marleen Praet

**Affiliations:** Laboratory of Anatomopathology, General Reference Hospital of Kinshasa, Kinshasa, Democratic Republic of the Congo; N.Goormaghtigh Institute of Pathology, Ghent University Hospital, De Pintelaan 185, Ghent, Belgium; Institute of Tropical Medicine, Nationalestraat 155, Antwerp, Belgium; Department of Microbiology, Clinical Chemistry and Immunology, Ghent University Hospital, De Pintelaan 185, Ghent, Belgium; Faculty of Medicine and Life Sciences, Hasselt University, Agoralaan Building D, Diepenbeek, Belgium; International Centre for Reproductive Health, Ghent University Hospital, De Pintelaan 185, Ghent, Belgium

**Keywords:** Cervical intraepithelial neoplasia, Human papillomavirus, Risk factors, Cross-sectional studies, Democratic Republic of the Congo

## Abstract

**Background:**

Cancer of the uterine cervix is the leading cause of cancer-related death among women in Sub-Saharan Africa, but information from the Democratic Republic of the Congo (DRC) is scarce. The study objectives were to: 1/ assess prevalence of (pre)cancerous cervical lesions in adult women in Kinshasa, 2/ identify associated socio-demographic and behavioural factors and 3/ describe human papillomavirus (HPV) types in cervical lesions.

**Methods:**

A cross-sectional study was conducted in Kinshasa. Between 2006 and 2013, four groups of women were recruited. The first two groups were included at HIV screening centres. Group 1 consisted of HIV-positive and group 2 of HIV-negative women. Group 3 was included in large hospitals and group 4 in primary health centres. Pap smears were studied by monolayer technique (Bethesda classification). Low- or high-grade squamous intraepithelial lesions or carcinoma were classified as LSIL+. HPV types were determined by INNO-LiPA®. Bivariate and multivariable analyses (logistic regression and generalised estimating equations (GEE)) were used to assess associations between explanatory variables and LSIL+.

**Results:**

LSIL+ lesions were found in 76 out of 1018 participants. The prevalence was 31.3 % in group 1 (*n* = 131 HIV-positive women), 3.9 % in group 2 (*n* = 128 HIV-negative women), 3.9 % in group 3 (*n* = 539) and 4.1 % in group 4 (*n* = 220). The following variables were included in the GEE model but did not reach statistical significance: history of abortion, ≥3 sexual partners and use of chemical products for vaginal care. In groups 3 and 4 where this information was available, the use of plants for vaginal care was associated with LSIL+ (adjusted OR 2.70 (95 % confidence interval 1.04 – 7.01). The most common HPV types among HIV-positive women with ASCUS+ cytology (ASCUS or worse) were HPV68 (12 out of 50 samples tested), HPV35 (12/50), HPV52 (12/50) and HPV16 (10/50). Among women with negative/unknown HIV status, the most common types were HPV52 (10/40), HPV35, (6/40) and HPV18 (5/40).

**Conclusion:**

LSIL+ lesions are frequent among women in Kinshasa. The use of plants for vaginal care deserves attention as a possible risk factor for LSIL+. In this setting, HPV16 is not the most frequent genotype in samples of LSIL+ lesions.

## Background

Cervical cancer constitutes a major health problem worldwide. It is responsible for 530,000 new cases of cancer and causes 270,000 deaths each year [1, 2]. Up to 80–85 % of cervical cancer-related deaths occur in low-income countries [1, 3]. In African women, it is the second most common cancer after breast cancer with an incidence rate of about 25 per 100,000 women per year. In Sub-Saharan Africa the incidence rate amounts to about 30–35, and here it is the most frequent cancer in women (for African data see [3–6]). It is expected, even on demographic grounds, that the burden of cervical cancer will further increase in Africa over the next years [7]. In contrast, in high-income countries such as the US and Europe the age-standardized incidence rate is about 6 to 10 per 100,000 women per year [1, 2]. Also the number of deaths from cervical cancer is nearly ten times lower in high-income countries [8, 9]. Differences between low- and high-income countries have been related to differences in exposure to risk factors and adequacy of screening. The most important risk factor is human papillomavirus (HPV) infection. Several other factors have been found to increase the risk of cervical cancer, possibly through their relation with the risk of HPV infection: number of sexual partners, early sexual activity [10], parity [11], long-term use of oral contraceptives [12–14], smoking [15] and HIV/AIDS [16–18].

The link between cervical cancer and HPV infection has been well established [16, 19–22]. From the more than 100 types of HPV described, about 40 are known to infect the genital tract and about 20 have been classified as oncogenic to humans [23–25]. Persistent infection with high-risk HPV has been considered as the necessary condition for malignant transformation of the cervical epithelium. In most studies, HPV16 and HPV18 are the predominant genotypes: they cause about 70 % of precancerous lesions and cervical cancer [26]. In Sub-Saharan Africa however, other oncogenic genotypes have been reported [22, 27–32].

In most Sub-Saharan countries, data on the prevalence and mortality of cervical cancer are either sparse or unavailable. Only 17 % of African countries have a national programme and a specific budget for fighting cervical cancer. And where such a cervical cancer programme exists, the effective coverage may be low. In addition, those women at the highest risk of developing cervical cancer may have the most difficult access to care [33, 34]. The prevalence of precancerous and cancerous lesions has been studied in a small number of women in the Democratic Republic of the Congo (DRC) [35–40]. Some of these reports include the prevalence of HPV and/or cervical lesions in HIV-positive women [35, 40].

Because of the high burden of cervical cancer in Sub-Saharan Africa, and presumably also in the DRC, the primary objectives of this study were to: 1/ evaluate the prevalence of (pre)cancerous lesions, 2/ identify associated socio-demographic and behavioural factors and 3/ describe HPV types present among women in Kinshasa.

## Results

### Characteristics of study participants

The total number of participants was 1018. One hundred thirty-one HIV-positive women were recruited at HIV screening centres (group 1); 128 HIV-negative women came from the same HIV screening centres (group 2); 539 women were recruited at large hospitals (group 3); and 220 were referred through small health centres (group 4). The HIV status of the women in groups 3 and 4 was unknown.

Table 1 shows the age and age-related characteristics of the participants. The mean age for all participants was 43.0 years (±12.8 standard deviation (SD)). The mean age of menarche was 14.3 years (±1.9) and the age of the first sexual intercourse was 18.5 years (±3.9). Table 2 summarises the socio-demographical characteristics of the study population. About half of the women were married, with the highest percentage in group 3 (61.2 %) and the lowest in group 2 (20.3 %). About one in six women was widowed (18.0 %), and one in four was single. Concerning pregnancies and parity, 44.0 % of the women reported six or more pregnancies, 29.8 % responded to have six or more children and 60.7 % reported to have had an abortion (not specified whether spontaneously or not).

**Table 1:** Age-related characteristics of the study population

	Group 1 (*n* = 131)	Group 2 (*n* = 128)	Group 3 (*n* = 539)	Group 4 (*n* = 220)	Total (*n* = 1018)
	HIV screening centres	HIV screening centres	Hospitals	Health centres	
	HIV-positive women	HIV-negative women	HIV status unknown	HIV status unknown	
Age					
Mean (years) ± SD	38.8 ± 9.1	29.4 ± 7.9	45.2 ± 11.0	48.0 ± 14.8	43.0 ± 12.8
Range (years)	20 – 59	17 - 51	20 – 76	20 – 82	17 - 82
*n* with available data	131	128	537	216	1012
Age of menarche					
Mean (years) ± SD	15.0 ± 1.9	14.5 ± 1.5	14.2 ± 1.9	14.5 ± 2.0	14.3 ± 1.9
Range (years)	11 – 20	12- 17	10 – 21	10 - 20	10 - 21
*n* with available data	50	26	502	208	786
Age of first sexual intercourse					
Mean (years) ± SD	17.2 ± 2.5	18.0 ± 3.2	18.8 ± 3.9	19.1 ± 4.5	18.5 ± 3.9
Range (years)	12 – 25	12 – 32	10 – 36	12 – 32	10 – 36
*n* with available data	127	125	523	207	982

*n*: number of participants with available data in each group; SD: standard deviation

**Table 2:** Socio-demographic characteristics of the study population

	Group 1 (*n* = 131)		Group 2 (*n* = 128)		Group 3 (*n* = 539)		Group 4 (*n* = 220)		Total (*n* = 1018)	
	HIV screening centres		HIV screening centres		Hospitals		Health centres			
	HIV-positive women		HIV-negative women		HIV status unknown		HIV status unknown			
	*n*	%	*n*	%	*n*	%	*n*	%	*n*	%
Marital status	130		128		533		217		1008	
Married	43	(33.1)	26	(20.3)	326	(61.2)	120	(55.3)	515	(51.1)
Single	39	(30.0)	95	(74.2)	76	(14.3)	33	(15.2)	243	(24.1)
Widowed	34	(26.2)	4	(3.1)	92	(17.3)	51	(23.5)	181	(18.0)
Divorced	14	(10.8)	3	(2.3)	39	(7.3)	13	(6.0)	69	(6.9)
Formal employment	129		126		525		215		995	
No	73	(56.6)	67	(53.2)	225	(42.9)	109	(50.7)	474	(47.6)
Yes	56	(43.4)	59	(46.8)	300	(51.7)	106	(49.3)	521	(52.4)
Number of pregnancies	128		126		517		219		990	
0-2	29	(22.7)	87	(69.1)	131	(25.3)	53	(24.2)	300	(30.3)
3-5	42	(32.8)	28	(22.2)	124	(24.0)	60	(27.4)	254	(25.7)
6 or more	57	(44.5)	11	(8.7)	262	(50.7)	106	(48.4)	436	(44.0)
Number of childbirths	128		126		520		218		992	
0-2	48	(37.5)	100	(79.4)	190	(36.5)	70	(32.1)	408	(41.1)
3-5	41	(32.0)	20	(15.9)	161	(31.0)	66	(30.3)	288	(29.0)
6 or more	39	(30.5)	6	(4.8)	169	(32.5)	82	(37.6)	296	(29.8)
Abortion	128		126		518		218		990	
No	48	(37.5)	61	(48.4)	175	(33.8)	105	(48.2)	389	(39.3)
Yes	80	(62.5)	65	(51.6)	343	(66.2)	113	(51.8)	601	(60.7)

*n*: absolute number; %: percentage of participants in each study group and each category

Behaviour-related characteristics of the study population are shown in Table 3. About half of the participants reported to have had zero to two lifetime sexual partners. In groups 1 and 2 (recruited at HIV screening centres), more women reported to have had three or more sexual partners (65.4 % and 61.2 % respectively). One out of five women declared to have used hormonal contraceptives (19.2 %); no information is available on the duration of its use. One out of four women reported alcohol consumption, including regular as well as irregular use. About the same percentage (26.0 %) reported to use chemical products for vaginal care. The women in groups 3 and 4 were also asked about the use of plants for vaginal care: 11.4 % of the women confirmed the intravaginal application of plants or vegetable products. Although the structured interview did not include specific questions about the type of plants that were used, some interviewers took note of what some of the women reported. Terms that were mentioned repeatedly in the local language Lingala included: mbonzi-mbonzi (leaves of a tree), tangawisi (ginger), tomate (tomato), lumba-lumba (medicinal leaves) and ngai-ngai (sorrel).

**Table 3:** Behaviour-related characteristics of the study population

	Group 1 (*n* = 131)		Group 2 (*n* = 128)		Group 3 (*n* = 539)		Group 4 (*n* = 220)		Total (*n* = 1018)	
	HIV screening centres		HIV screening centres		Hospitals		Health centres			
	HIV-positive women		HIV-negative women		HIV status unknown		HIV status unknown			
	*n*	%	*n*	%	*n*	%	*n*	%	*n*	%
Number of sexual partners	129		127		529		190		975	
Zero to two	50	(38.8)	44	(34.7)	304	(57.5)	132	(69.5)	530	(54.4)
Three or more	79	(61.2)	83	(65.4)	225	(42.5)	58	(30.5)	445	(45.6)
Use of hormonal contraception	131	131	128		539		171		969	969
No	105	(80.2)	118	(92.2)	421	(78.1)	139	(81.3)	783	(80.8)
Yes	26	(19.8)	10	(7.8)	118	(21.9)	32	(18.7)	186	(19.2)
Alcohol consumption	131		127		529		138		925	925
No	123	(93.9)	99	(78.0)	378	(71.5)	87	(63.0)	687	(74.3)
Yes	8	(6.1)	28	(22.1)	151	(28.5)	51	(37.0)	238	(25.7)
Use of plants for vaginal care					539		212		751	751
No					513	(95.2)	153	(72.2)	666	(88.6)
Yes					26	(4.8)	59	(27.8)	85	(11.4)
Use of chemical products for vaginal care	131		128		539		151		949	
No	98	(74.8)	92	(71.9)	445	(82.6)	67	(44.4)	702	(74.0)
Yes	33	(25.2)	36	(28.1)	94	(17.4)	84	(55.6)	247	(26.0)

*n*: absolute number; %: percentage of participants in each study group and each category

### Prevalence of low-grade squamous intraepithelial lesions or worse (LSIL+)

In total, 76 of 1018 women were diagnosed with LSIL+ lesions (i.e. low- or high-grade squamous intraepithelial lesions (LSIL or HSIL) or invasive cancer, Table 4). Among the HIV-positive women of group 1, the prevalence of LSIL+ was 31.3 % (95 % confidence interval (CI): 24.0 % – 39.7 %). Among the HIV-negative women recruited in the same clinics (group 2), the LSIL+ prevalence was 3.9 % (95 % CI: 1.7 % – 8.8 %). Among the participants coming from the large hospitals (group 3), the prevalence was 3.9 % (95 % CI: 2.6 % – 5.9 %) and among the women coming via smaller health centres (group 4), 4.1 % (95 % CI: 2.2 % – 7.6 %). Among the women with unknown or negative HIV status, lesions classified as ASCUS (atypical squamous cells of undetermined significance) or ASC-H (atypical squamous cells, cannot rule out high-grade lesion) were more frequent than LSIL+ lesions (Table 4).

**Table 4:** Numbers and proportions of different lesions according to the Bethesda 2001 classification, per study group

	Group 1 (*n* = 131)	Group 2 (*n* = 128)	Group 3 (*n* = 539)	Group 4 (*n* = 220)	Total (*n* = 1018)
	HIV screening centres	HIV screening centres	Hospitals	Health centres	
	HIV-positive women	HIV-negative women	HIV status unknown	HIV status unknown	
	*n* (%)	*n* (%)	*n* (%)	*n* (%)	1018 (%)
NILM	80 (61.1)	117 (91.4)	449 (83.3)	188 (85.5)	834 (81.9)
ASCUS	9 (6.9)	5 (3.9)	46 (8.5)	19 (8.6)	79 (7.8)
ASC-H	1 (0.8)	1 (0.8)	23 (4.3)	4 (1.8)	29 (2.9)
LSIL	2 (1.5)	0 (0.0)	0 (0.0)	0 (0.0)	2 (0.2)
HSIL	30 (22.9)	5 (3.9)	17 (3.2)	5 (2.3)	57 (5.6)
Ca	9 (6.9)	0 (0.0)	4 (0.7)	4 (1.8)	17 (1.7)
Subtotal: LSIL+	41 (31.3)	5 (3.9)	21 (3.9)	9 (4.1)	76 (7.5)

NILM negative for intraepithelial lesion and malignancy

ASCUS atypical squamous cells of undetermined significance

ASC-H atypical squamous cells, cannot rule out high-grade lesion

LSIL low-grade squamous intraepithelial lesion

HSIL high-grade squamous intraepithelial lesion

Ca Carcinoma

LSIL+ cytology findings compatible with (pre)cancerous lesions (includes low- and high-grade squamous intraepithelial lesions and carcinoma)

### Socio-demographic factors and behaviour characteristics associated with LSIL+ lesions

In each of the four groups, bivariate associations were assessed between all explanatory factors (age, socio-demographic and behaviour-related characteristics) and the presence of LSIL+ lesions. Crude odds ratios (ORs) are given in Table 5. Explanatory factors with a *P*-value of <0.2 were included in a model of multiple logistic regression (one model per study group). The adjusted ORs are given in Table 6. None of these associations reached statistical significance on multiple logistic regression.

**Table 5:** Bivariate associations between socio-demographic and behavioural characteristics and presence of LSIL+ lesions, per study group

		Group 1 (*n* = 131)		Group 2 (*n* = 128)		Group 3 (*n* = 539)		Group 4 (*n* = 220)	
		HIV screening centres		HIV screening centres		Hospitals		Health centres	
		HIV-positive women		HIV-negative women		HIV status unknown		HIV status unknown	
		Crude OR	95 % CI	Crude OR	95 % CI	Crude OR	95 % CI	Crude OR	95 % CI
Age (in years)^a^		0.99	0.95 - 1.03	1.11**	1.00 - 1.24	1.02	0.98 - 1.07	1.02	0.97 - 1.06
Age of menarche (in years)^a^		0.94	0.67 - 1.30	2.87	0.36 - 23.07	1.03	0.82 - 1.30	0.80	0.55 - 1.15
Age of first sexual intercourse		0.89*	0.76 - 1.05	1.09	0.86 - 1.38	1.01	0.90 - 1.13	0.84*	0.68 - 1.06
Marital status	married	1.00		1.00*		1.00		1.00	
	single	0.56	0.21 - 1.48	0.82	0.08 - 8.18	2.23	0.74 - 6.71	1.23	0.24 - 6.38
	widowed	1.16	0.45 - 2.94	8.33	0.41 - 170.67	1.44	0.44 - 4.69	§	
	divorced	0.75	0.20 - 2.79	§		1.71	0.36 - 8.10	1.58	0.18 - 14.28
Formal employment		0.75	0.35 - 1.61	1.74	0.28 - 10.79	1.92*	0.73 - 5.03	0.28*	0.06 - 1.38
Number of pregnancies	0 - 2	1.00		1.00**		1.00		1.00	
	3 - 5	1.31	0.47 - 3.70	1.96	0.14 - 18.04	1.06	0.33 - 3.38	0.28	0.03 - 2.80
	6 or more	1.31	0.49 - 3.51	10.01	1.18 - 75.36	0.66	0.22 - 1.93	0.83	0.19 - 3.59
Number of childbirths	0 - 2	1.00*		1.00**		1.00		1.00	
	3 - 5	1.08	0.96 - 5.77	8.65	1.34 - 55.65	0.31	0.15 - 1.70	0.70	0.11 - 4.31
	6 or more	1.18	0.45 - 3.06	§		1.00	0.38 - 2.65	1.15	0.25 - 5.30
Abortion		1.70*	0.77 - 3.78	1.43	0.23 - 8.85	2.09*	0.69 - 6.35	1.17	0.31 - 4.48
Three or more lifetime sexual partners		1.26	0.58 - 2.74	2.18	0.24 - 20.10	2.27*	0.92 - 5.57	0.91	0.17 - 4.82
Hormonal contraception		1.21	0.49 - 3.00	3.17	0.32 - 31.40	1.12	0.40 - 3.12	§	
Alcohol consumption		0.30	0.04 - 2.49	2.46	0.39 - 15.51	1.57	0.64 - 3.87	4.62*	0.86 - 24.75
Plants for vaginal care		†		†		3.59*	0.99 – 13.05	2.15	0.56 – 8.31
Chemicals for vaginal care		0.63	0.26 - 1.55	§*		0.49	0.11 - 2.13	2.06	0.39 - 10.95

LSIL+: cytology findings compatible with (pre)cancerous lesions (includes low- and high-grade squamous intraepithelial lesions and carcinoma)

OR odds ratio; 95 % CI 95 % confidence interval of the odds ratio

^a^Age was treated as a continuous variable. Interpretation, e.g. in group 2: the odds of LSIL+ lesions increased with a factor 1.11 for each one-year increase in age

**P-value of Wald, chi-squared or Fisher exact test < 0.05

*P-value of Wald, chi-squared or Fisher exact test is not significant but is less than 0.2

§Odds ratio could not be calculated because there were cells without observations

†Data about use of plants for vaginal care were not available in groups 1 and 2

**Table 6:** Multivariable evaluations of the association between explanatory variables and the presence of LSIL+ lesions

Type of analysis	Study groups included	Number of observations*	Explanatory variables included in the model	Adjusted OR	95 % CI
Logistic regression	1 (HIV screening centres HIV-positive women)	124	Age of first sexual intercourse^a^	0.86	0.72 – 1.02
			Number of pregnancies		
			0 – 2	1	
			3 – 5	0.95	0.31 – 2.86
			6 or more	0.83	0.28 – 2.44
			Abortion	1.98	0.83 – 4.75
Logistic regression	2 (HIV screening centres HIV-negative women)	123	Age^a^	1.11	0.95 – 1.31
			Marital status		
			married	1	
			single	5.26	0.27 – 102.32
			widowed	4.34	0.18 – 105.99
			divorced	§	
			Number of pregnancies		
			0 – 2	1	
			3 – 5	1.15	0.07 – 18.34
			6 or more	4.27	0.22 – 81.82
Logistic regression	3 (Hospitals HIV status unknown)	502	Formal employment	2.08	0.74 – 5.88
			Abortion	1.65	0.53 – 5.14
			≥3 lifetime sexual partners	1.75	0.69 – 4.46
			Plants for vaginal care	2.85	0.75 – 10.82
Logistic regression	4 (Health centres HIV status unknown)	203	Age of first sexual intercourse^a^	0.86	0.68 – 1.07
			Formal employment	0.30	0.06 – 1.50
GEE	1, 2 3 and 4	886	Abortion	1.60	0.97 – 2.63
			≥3 lifetime sexual partners	1.29	0.83 – 1.99
			Chemicals for vaginal care	0.65	0.37 – 1.14
Logistic regression	3 and 4	643	Alcohol consumption	1.76	0.80 – 3.86
			≥3 lifetime sexual partners	1.58	0.72 – 3.46
			Plants for vaginal care	2.70	1.04 – 7.01**

LSIL+: cytology findings compatible with (pre)cancerous lesions (includes low- and high-grade squamous intraepithelial lesions and carcinoma)

OR odds ratio; 95 % CI 95 % confidence interval of the odds ratio

GEE: generalized estimating equations (population-averaged model; group variable: study group)

^a^Age was treated as a continuous variable

§ Odds ratio could not be calculated because there were cells without observations

**P-value of Wald test < 0.05

All participants were then analysed together using generalised estimating equations (GEE) to account for clustering within the study groups. Also in the GEE analysis, all factors with a *P*-value <0.2 on bivariate evaluation were included in a multivariable model. The final GEE model (one model for all the study groups) included history of abortion (adjusted OR 1.60; 95 % CI 0.97 – 2.63), more than three sexual partners (adjusted OR 1.29; 95 % CI 0.83 – 1.99) and use of chemical products for vaginal care (adjusted OR 0.65; 95 % CI 0.37 – 1.14). Adding age to the model did not substantially change the ORs. None of the associations in this model reached statistical significance (Table 6).

The use of plants for vaginal care could only be evaluated in groups 3 and 4 because this information was not available for the women in the other groups. In a multiple logistic regression model including the use of plants, alcohol consumption and having had three or more sexual partners, the adjusted OR for the association between the use of plants for vaginal care and the presence of LSIL+ lesions was 2.70 (95 % CI 1.04 – 7.01; Table 6).

### Determination of HPV DNA and HPV typing

The following cytology results were classified as ASCUS+ and the corresponding samples were submitted for HPV typing: ASCUS, ASC-H, LSIL, HSIL or carcinoma. In the HIV-positive group, 50 out of the 52 ASCUS+ samples contained HPV DNA. HPV16 was found in 10 out of 50 samples, and HPV18 in 5 (Table 7). Other genotypes that were frequently detected were HPV35 (*n* = 12), HPV52 (*n* = 12), HPV68 (*n* = 12), HPV51 (*n* = 10), and HPV31 (*n* = 9). In the groups of women with negative or unknown HIV status, 50 samples were tested with INNO-LiPA of which 40 contained detectable HPV DNA (Table 7). Here, the most frequently detected genotypes were HPV52 (*n* = 10), HPV35 (*n* = 6), HPV16 (*n* = 3), HPV18 (*n* = 5), HPV51 (*n* = 5), and HPV54 (*n* = 5). In addition to the three samples in which HPV16 was detected through the INNO-LiPA test, there were three samples in which both the Abbot Real Time and the GenoID test indicated the presence of HPV16.

**Table 7:** HPV genotyping results for women with ASCUS+ cytology

HPV genotype	Group 1 (HIV-positive women)	Groups 2, 3 and 4 (women with unknown or negative HIV status)
	Number of women with ASCUS + = 52	Number of women with ASCUS + = 133
	Number of samples in which HPV typing results were available = 50	Number of samples in which HPV typing results were available = 40
6	3 (6 %)	0 (0 %)
11	2 (4 %)	1 (3 %)
16	10 (20 %)	3 (8 %)^a^
18	5 (10 %)	5 (13 %)
31	9 (18 %)	2 (5 %)
33	6 (12 %)	4 (10 %)
35	12 (24 %)	6 (15 %)
39	6 (12 %)	4 (10 %)
40	1 (2 %)	1 (3 %)
43	2 (4 %)	0 (0 %)
44	5 (10 %)	0 (0 %)
45	7 (14 %)	2 (5 %)
51	10 (20 %)	5 (13 %)
52	12 (24 %)	10 (25 %)
53	5 (10 %)	1 (3 %)
54	3 (6 %)	5 (13 %)
56	6 (12 %)	3 (8 %)
58	7 (14 %)	0 (0 %)
59	2 (4 %)	1 (3 %)
66	7 (14 %)	4 (10 %)
68	12 (24 %)	3 (8 %)
69	1 (2 %)	1 (3 %)
70	5 (10 %)	1 (3 %)
74	4 (8 %)	4 (10 %)
82	0 (0 %)	1 (3 %)

HPV human papillomavirus

ASCUS+ includes: atypical squamous cells of undetermined significance (ASCUS); atypical squamous cells; cannot rule out high-grade lesion (ASC-H); low- grade squamous intraepithelial lesions (LSIL), high-grade squamous intraepithelial lesions (HSIL) and carcinoma

^a^In addition to the three samples in which HPV16 was detected through the INNO-LiPA test, there were three samples in which both the Abbot Real Time and the GenoID test indicated the presence of HPV16

In groups 2, 3 and 4, 55 % of samples which tested positive for HPV DNA contained one single HPV type. Two genotypes were found in 23 % of the samples, three genotypes in 9 %, four in 7 % and more than four in 7 % of HPV-DNA positive samples. In the HIV-positive group (group 1), a single HPV infection occurred only in 20.0 % of the samples. Two genotypes were found in 38 %, three in 9 %, four in 16 % and more than four in 18 % of the samples.

## Discussion

The present study was performed to assess the prevalence of LSIL+ lesions and to identify associated factors in different groups of women in Kinshasa. The prevalence of LSIL+ lesions ranged from approximately 4 % in women with unknown or negative HIV status to 31 % in HIV-positive women. We found an association between the practice of intravaginal insertion of plants and the presence of LSIL+. HPV types 16 and 18 which are known to cause cervical cancer in many countries worldwide appear to be less predominant in women in Kinshasa.

The prevalence of LSIL+ lesions that we found in the current study in women with unknown or seronegative HIV status (4 %) is consistent with the few studies previously performed in Kinshasa (3 % and 5 %) [35, 36] and in Bukavu in the eastern part of the country (7 %) [37]. Numbers of the same order of magnitude have been published in other Sub-Saharan countries. A prevalence between 4 and 10 % was found in studies in Burkina-Faso, Nigeria, Tanzania, South Africa, Malawi and Kenya [29, 41–49]. Higher prevalences (16 %) were reported in studies in the Central African Republic and Uganda [50, 51]. These reports and our findings highlight the high and heterogeneous frequency of (pre) cancerous lesions in different countries of Sub-Saharan Africa. Furthermore, in our study, lesions classified as ASCUS and ASC-H were also frequent (more frequent than LSIL+) among women with negative or unknown HIV status.

Cervical cancer is known to be more frequent among HIV-positive women. In the DRC, it is estimated that 1.9 % of the adult women are HIV infected, with differences between women living in urban areas (2.4 %) and rural areas (1.0 %) [52]. We found a prevalence of LSIL+ lesions of 31 % in HIV-seropositive women. This finding is consistent with an earlier result (27 %) described in a small group of seropositive women in Kinshasa [35]. Also in other Sub-Saharan countries (pre)cancerous lesions were about five times more frequent in HIV-positive than in HIV-negative women [41, 42, 45, 48–51, 53].

Several demographic, economical and behavioural risk factors have been studied in relation to cervical cancer. Most of them may influence the risk of cancer through their effects on the risk of HIV and HPV infection. In the current study, we found a significant association between the intravaginal application of plant products and the presence of LSIL+ lesions. It is a frequent practice in Sub-Saharan Africa to use herbs, leaves and bark of trees to reduce vaginal lubrication and increase friction during sexual intercourse (dry sex) [54–59]. The perception is that dry sex increases sexual enjoyment. A study in the DRC revealed that one third of the women had used intra-vaginal substances at some time [55]. Another Congolese study looked into how specific plants are used and what the chemical and microbiological consequence of this traditional practice could be [60].

Most of the studies about the vaginal use of plants have been done in relation to the risk of HIV infection. It has been hypothesised that differences in the vaginal environment may partially explain the different HIV transmission probabilities that are observed across populations [61]. Similar mechanisms may play a role in transmission and clearance of HPV [62–64]. The insertion of plant products and the increased friction during dry sex may alter the vaginal microbiota and cause traumatic microlesions in the vaginal wall facilitating the entry of HPV [62–64].

The association between HPV infection and cervical cancer is well established, but the specific HPV genotypes that are involved in neoplasia differ across populations. The HPV types that have been most frequently linked to cervical cancer are: 16, 18, 31, 33, 45, 52 and 58 [53, 65]. HPV16 and 18 are responsible for 70 % of precancerous lesions and cervical cancer worldwide and consequently, these are the HPV types which the vaccine development has focused on. Nevertheless, our study suggests that HPV16 and 18 are less frequent (maximally 30 % of women with ASCUS+) and that HPV types 35, 52 and 68 are more predominant than in other regions. Other studies in the DRC have also described specific patterns of HPV types [38, 40].

In other Sub-Saharan countries, the frequency of HPV16 and 18 varies. Some countries have reported patterns that resemble the situation in Europe and the United States [22, 48, 66, 67], whereas in other countries, HPV types other than 16 and 18 appear to be more prominent [29, 47, 68, 69].

The DRC is a large country that is facing many complex problems at the same time. As a consequence, cervical cancer is not getting the attention that would be required for adequate disease control. Yet, the burden caused by cervical cancer in the DRC would justify a coordinated control strategy. This study together with a previous report illustrates that women in Kinshasa are willing to participate in prevention and control activities [70]. Interventions that could help to reduce the morbidity and mortality of cervical cancer include vaccination for HPV, systematic screening and early treatment. The effectiveness of such interventions may benefit from further research into the epidemiology of oncogenic HPV types and modifiable risk factors such as the use of plants for intimate care.

## Conclusion

Our work illustrates that the prevalence of (pre)cancerous lesions in women from different districts in Kinshasa is approximately 4 %. In HIV-positive women, the prevalence is about eight times higher. Traditional practices concerning vaginal hygiene may increase the risk of malignant transformation. More extensive studies, including rural areas, are needed to unravel the contribution of different HPV types in the development of cervical cancer.

## Methods

### Study design, setting and participants

We performed a cross-sectional study on the prevalence of precancerous and cancerous lesions of the uterine cervix in four groups of women in Kinshasa. The first two groups consisted of women who participated in a voluntary screening programme for HIV in the ACS/AMO-CONGO centre (Action Communautaire contre le Sida/Avenir Meilleur pour les Orphelins du Sida au Congo) and in the Centre Hospitalier Monkole 3. Women who visited these centres for HIV screening and care between September 2006 and January 2007 were invited to participate in free cervical cancer screening. The first group (*n* = 131) consisted of women who were found to be seropositive for HIV; the second group (*n* = 128) consisted of HIV-seronegative women. The diagnosis of HIV was based on two rapid tests (Determine®HIV-1/2 (Abbott) and OraQuick®HIV-1/2 (OraSure Technologies)) combined with one of the following serological tests: Vironostika® Uni-Form II Plus O (BioMérieux), Enzygnost® Anti-HIV ½ Plus (Siemens), or Inno-Lia HIVI/II Score (Inno-LIA®, Innogenetics). The third group (*n* = 539) consisted of women who consulted the gynaecology department of the Provincial Reference Hospital of Kinshasa (Hôpital Provincial Général de Référence de Kinshasa, HPGRK) and the Ngaliema hospital. Both hospitals are large state hospitals in the centre of the city. Data were collected from July to August 2009. At that time, a sensitisation campaign for free cervical screening was broadcast on television, posters advertising cervical cancer screening were shown at the hospitals, and a symposium on cervical cancer took place at the HPGRK. The participants of the fourth group (*n* = 220) were recruited via primary health care centres in the communities of Kimbanseke, Kisenso, Ndjili and Lemba, located in the poorer suburbs of the city. Data were collected from September 2012 to January 2013 after a campaign in local churches for free cervical cancer screening.

All women older than 17 who were willing to participate in the study and for whom a liquid-based cytology (LBC) result was available were included in the analysis.

### Variables, data sources and measurement

#### Cytology

Cytology was the main outcome variable. Cervical smears were collected with Cervex Brush and conserved in ThinPrep solution for LBC and HPV typing. Samples were kept at 4 °C. ThinPrep vials were transferred to the Pathology Laboratory of the University Hospital of Ghent, Belgium, for further analysis. The collected smears were independently read and interpreted by two pathologists. The pathologists were not aware of the HIV and HPV status at the time of reading the microscopy slides. In case of discrepancy, the slides were reread by both pathologists for a final interpretation. The cytology results were reported according to the Bethesda Classification 2001 of cervical pathology. Women with cytology results indicating LSIL (low-grade squamous intraepithelial lesion), HSIL (high-grade squamous intraepithelial lesion) and invasive carcinoma were considered to have precancerous or cancerous lesions and classified as LSIL+ (according to [71]). Women with ASCUS (atypical squamous of undetermined significance), ASC-H (atypical squamous, cannot rule out high-grade lesion) results or worse (ASCUS+) underwent HPV-DNA determination. Women with NILM (negative for intraepithelial lesion and malignancy) or inflammation were considered to be free of cancer.

#### Determination of HPV DNA and HPV typing

HPV typing was done in participants with ASCUS+ cytology results. Samples from women with ASCUS and ASC-H were included in the HPV evaluation as the European guidelines recommend to determine HPV DNA in these groups. INNO-LiPA HPV Genotyping Extra (Innogenetics, Zwijnaarde, Belgium) was used for HPV typing. It is molecular technique based on the principle of reverse hybridisation designed to recognize fifteen high-risk HPV types (16, 18, 31, 33, 35, 39, 45, 51, 52, 56, 58, 59, 68, 73 and 82), three probable high-risk HPV types (26, 53 and 66), seven low-risk HPV types (6, 11, 40, 43, 44, 54 and 70) and three non-classified HPV types (69, 71 and 74).) The testing strategies varied across the study groups. In groups 1 and 2, INNO-LiPA Genotyping was done for all ASCUS+ cases. In groups 3 and 4, the INNO-LiPA test, due to its high cost, was only done if two other tests gave discordant results (Abbott Real Time High Risk HPV test (Abbott, Madison, USA) and Full Spectrum PCR HPV Amplification and Detection/Genotyping System (GenoID Molecular Diagnostics Laboratory, Budapest, Hungary)). We present all available INNO-LiPA results because this test detects many different HPV types [72]. Concordant Abbott and GenoID results for HPV16 and HPV18 are also reported.

#### Socio-economic, gynaecological/medical and behaviour variables

Socio-economic and behaviour characteristics that were studied in association with the presence or absence of LSIL+ were: age, age of menarche, age of first sexual contact, marital status, formal employment, life-time number of sexual partners, use of products for vaginal care (chemicals and products from plants), alcohol consumption (no alcohol *versus* any consumption), number of pregnancies, number of childbirths, and history of abortion (without discrimination between spontaneous or provoked). Concerning the practice of intravaginal application of plants, information was only available for groups 3 and 4.

The variables “age”, “age of menarche” and “age of first sexual contact” follow a relatively normal distribution and are presented and analysed as continuous variables. The variables “number of sexual partners”, “number of pregnancies” and “number of deliveries” follow a clearly abnormal distribution and are presented and analysed as categorical variables.

Information about the socio-economic situation and behaviour characteristics was obtained through a structured interview by medical doctors. Interviewers followed a training session before the start of the study.

### Statistical analysis

The prevalence of LSIL+ lesions with 95 % confidence intervals (95 % CI, Wilson score method without continuity correction) is reported for each of the four groups. Next, we used bivariate and multiple logistic regression to assess the association between socio-demographic and behaviour variables and the presence of LSIL+ lesions within each of the four groups. Finally, we analysed all participants together using generalized estimating equations (GEE) to account for clustering within the groups. The results of all logistic regression and GEE analyses are reported as odds ratios (OR) with 95 % confidence intervals (95 % CI). Data was missing for some participants in some of the explanatory variables; for each of the analyses, the number of included participants is given. We used Stata/IC 10.1 for data analysis.

### Ethical considerations

The study protocol on the collection of data and the reporting of data to participants was approved by the Ethics Committee of the School of Health of the University of Kinshasa. After having explained the objectives of the study, all study participants signed a document of informed consent.
